# Generation of Subdiffraction Optical Needles by Simultaneously Generating and Focusing Azimuthally Polarized Vortex Beams through Pancharatnam–Berry Metalenses

**DOI:** 10.3390/nano12224074

**Published:** 2022-11-19

**Authors:** Zhe Shen, Shijie Huang

**Affiliations:** School of Electronic and Optical Engineering, Nanjing University of Science and Technology, Nanjing 210094, China

**Keywords:** optical needle, Pancharatnam–Berry metalens, azimuthally polarized vortex beam

## Abstract

Needle beams have received widespread attention due to their unique characteristics of high intensity, small focal size, and extended depth of focus (DOF). Here, a single–layer all–dielectric metalens based on Pancharatnam–Berry (PB) was used to efficiently generate and focus an azimuthally polarized vortex beam at the same time. Then, additional phase or amplitude modulation was respectively adopted to work with the metalens to produce optical needles. By decorating the PB metalens with the binary optical element (BOE), an optical needle with full–width–at–half–maximum (FWHM) of 0.47 λ and DOF of 3.42 λ could be obtained. By decorating the PB metalens with an annular aperture, an optical needle with long DOF (16.4 λ) and subdiffraction size (0.46 λ) could be obtained. It is expected that our work has potential applications in super–resolution imaging, photolithography, and particle trapping.

## 1. Introduction

In recent years, the generation of tiny focal spots with high intensity has been an important research part in nanophotonics [1,2]. Focal spots with subdiffraction size and extended depth of focus (DOF), also called needle beams or optical needles, can be applied in super–resolution imaging [3], photolithography [4], and particle trapping [5]. Generally, the generation of needle beams mainly comes from the focusing of radially polarized beams (RPBs) [6,7]. Corresponding total focal fields are dominated by significantly enhanced longitudinal electric field components, resulting in strong longitudinally polarized optical needles. Compared with longitudinally polarized optical needles, subwavelength transversely polarized optical needles are especially popular in specific fields such as ultra-high density magnetic storage and atomic trapping [8]. Transverse polarization is usually associated with the focusing of an azimuthally polarized vortex beam rather than RPB. Hao et al. demonstrated that a sharper and purely transversely polarized focal spot could be obtained by focusing a phase encoded azimuthally polarized beam (APB), which is smaller than that of RPB or a linearly polarized beam (LPB) [9]. Transversely polarized needle beams can be further formed by highly focused azimuthally polarized vortex beams through phase elements [10], amplitude filters [11], or axicons [12]. Since the generation of azimuthally polarized vortex beams involves polarization and phase modulation, this means that it requires complex and bulky optical elements.

Metasurfaces can not only provide a new channel for manipulating the phase, amplitude, and polarization of incident light, but also reduce the thickness of optical devices, leading to miniaturized and integrated optical elements. Various optical elements from lenses [13], vortex plates [14] to polarization converted devices [15] have been demonstrated using metasurfaces. Metalens that possesses the main function of lens can also perform complete phase modulation. A polarization–insensitive metalens has been designed to obtain extended DOF of the focal spot and longitudinal high-tolerance imaging [16]. A needle beam with long DOF and subwavelength size by illuminating APB was generated through a polarization–insensitive metalens [17]. However, the production of pre-prepared vector beams requires complex optical elements. While metalenses based on Pancharatnam–Berry (PB) have the capability to control the polarization of light and subsequently produce vector beams [18,19]. These metalenses are based on polarization–dependent nanorods that locally act as wave plates (usually half−wave plates), but have different rotation angles on metalenses [19]. Moreover, PB metalenses can provide control of the polarization and phase simultaneously [20] where focused vector beams have been generated [21,22].

In this work, single-layer all-dielectric PB metalenses were used to simultaneously generate and focus azimuthally polarized vortex beams to form subdiffraction needle beams. The all−dielectric metalens was composed of TiO_2_ nanobricks and a SiO_2_ substrate. In addition to its high refractive index, TiO_2_ also has negligible absorption loss, which is contrary to the high loss caused by the use of silicon material metalens in the visible light band [13]. At present, such metasurfaces can be made using electron beam lithography and atomic layer deposition [23]. There will be some fabrication imperfections when fabricating the metasurface, resulting in slight changes in the structure. Capasso et al. [24] demonstrated that an imaging spatial resolution of about 200 nm was achieved by integrating liquid–immersed metalens into a commercial scanning confocal microscope. Even though the structure of the fabricated nanobrick was slightly changed, it still achieved the expected function, which indicates that the designed nanobrick had performance robustness against the fabrication imperfections. Based on the PB phase, simple LPBs, which are more readily available in the laboratory than vector beams, can be converted to APBs. According to the transmission phase, metalens can simultaneously control the discrete phases for vortex generation and light focusing. We first verified that the designed PB metalens could convert LPBs into azimuthally polarized vortex beams and focus simultaneously. Then, a binary optical element (BOE) or an annular aperture was used to combine with the metalens to generate optical needles. The obtained optical needles may apply to super-resolution imaging, photolithography, and particle trapping.

## 2. Theory and Designs of Metalens

### 2.1. Theoretical Study on Focusing the Azimuthally Polarized Vortex Beam

Under the illumination of the azimuthally polarized vortex beam (topological charge = 1), the electric field distributions near the focal field can be obtained by vector diffraction theory [25]:

(1) $$\left[\begin{array}{l} E_{r}\\ E_{\phi }\\ E_{z} \end{array}\right]=\left[\begin{array}{c} -Ae^{i\phi }(I_{0}+I_{2})\\ -iAe^{i\phi }(I_{0}-I_{2})\\ 0 \end{array}\right]$$

where

(2) $$I_{n}=\int_{0}^{\theta_{\max}}\cos^{1/2}\theta \sin\theta P(\theta )l_{0}(\theta )e^{ikz\cos\theta }J_{n}(k\cos\theta )d\theta$$

In Equation (1), *A* is a constant. In Equation (2), $\theta_{\max}=arcsin(NA)$ is the maximum angle of focus determined by the numerical aperture (NA, NA=sin[arctan(R/f)], where *R* is the radius of the metalens), and the value of NA is 0.9. *P*(*θ*) is the pupil function, Jn is the n order Bessel function of the first kind, k=2π/λ is the wave number, and *λ* is the corresponding incident wavelength. *l*_0_(*θ*) signifies the amplitude of the incident light at the pupil plane. For the planar wavefront, *l*_0_(*θ*) can be regarded as a constant *A*, while *l*_0_(*θ*) is the Bessel–Gaussian beam, which is given by:

(3) $$l_{0}(\theta )=\exp(-\beta^{2}{\left(\frac{\sin\theta }{\sin\theta_{\max}}\right)}^{2})J_{1}(2\beta \frac{\sin\theta }{\sin\theta_{\max}})$$

where *β* is the ratio of the pupil radius to the beam waist and set as 1.

**Figure 1:**
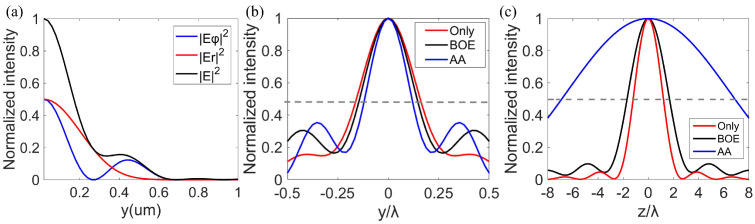
Theoretical calculation of the focal field of the azimuthally polarized vortex beam. (**a**) Refers to the radial normalized intensity distribution in the focal plane modulated only by the metalens. |E_φ_|^2^, |E_r_|^2^, and |E|^2^ represent normalized intensities of the azimuthal, radial, and total components, respectively. (**b**,**c**) Represent the normalized intensity profiles along the y−axis and z−axis, respectively. The gray dotted line indicates the position of the half maximum intensity. Only indicates the case of only the metalens. BOE and AA indicate the case of the phase of BOE and annular aperture working with the metalens, respectively.

It can be seen from Equation (1) that an additional radial electric field component is generated when the spiral phase is introduced. This is because polarization and phase are related, and the introduction of a phase difference in the incident wavefront results in a change in the polarized distribution. Due to this radial component, the polarization near the focal plane is spatially variable and complex (the polarization distribution related ellipticities of our generated needle beams were calculated as shown in the Supplementary Materials) and the polarization singularity at the center of the focal plane disappeared, which was similar to the bright and sharp focal spot of the RPB. As can be seen in Figure 1a, the electric field distributions of the focal spot of the azimuthally polarized vortex beam were theoretically calculated. The radial and azimuthal components constitute the total electric field in the focal plane. The focal fields of both the radial and azimuthal components possess bright focal spots, which are different from the doughnut-shaped focal spots of APBs with polarized singularities. As shown in Figure 1b,c, the full–width–at–half–maximum (FWHM) and DOF of the focal spot were 0.57 λ and 1.57 λ, respectively, which is close to the optical diffraction limit.

The BOE was introduced to improve the performance of the needle beam by enhancing the longitudinally polarized component of the focal field [6], so we used BOE to optimize the focal spot in order to generate an optical needle. When the phase distribution of BOE is loaded onto the metalens, *P*(*θ*) in Equation (2) is replaced by *P*(*θ*)*T*(*θ*), where the transfer function $T(\theta )=\exp(i\varphi_{BOE})$. The phase of the BOE *φ*_BOE_ exists only in the case of 0 or π, which appears alternately. We used a five-belt BOE, the corresponding phase distributions are as follows:

(4) $$\varphi_{BOE}=\left\{ \begin{array}{l} 0,\;\mathrm{for}\;0\leq \theta <\theta_{1},\;\theta_{2}\leq \theta <\theta_{3},\;\theta_{4}\leq \theta <\theta_{\max}\\ \pi ,\;\mathrm{for}\;\theta_{1}\leq \theta <\theta_{2},\;\theta_{3}\leq \theta <\theta_{4}\; \end{array}\right.$$

For four angles *θ_i_* (*i* = 1, 2, 3, 4), corresponding to four radial positions $r_{i}=\sin\theta_{i}/NA$, were optimized by the particle swarm optimization algorithm [26]. Here, the parameters of the BOE are given by:

(5) $$r_{1}=0.083,\;r_{2}=0.1659,\;r_{3}=0.2489,r_{4}=0.4348,\;\mathrm{and}\;r_{5}=1$$

Meanwhile, the upper and lower lines of the integral in Equation (2) change with *θ_i_*. The Bessel–Gaussian beam is incident, which means the corresponding amplitude *l*_0_(*θ*) is Equation (3). In Figure 1b,c, FWHM and DOF of the focal spot were 0.51 λ and 2.17 λ, respectively. Obviously, the BOE can help reduce the FWHM and prolong the DOF of the focal spot. In addition, an annular aperture also has a similar effect on the modulation of the focal spot [17,22]. After applying the annular aperture, the lower limit of Equation (2) becomes *θ*_min_ and the incident light is a plane wave. When the annular aperture σ = 0.9 (σ represents the ratio of the radius of the opaque gold plate to *R*, *θ*_max_ = arcsin(0.9), *θ*_min_ = 0.9*θ*_max_), the FWHM and DOF of the focal spot reached 0.42 λ and 15 λ, respectively, both of which were significantly improved compared with the results of the lens only.

### 2.2. Design of Metalens

**Figure 2:**
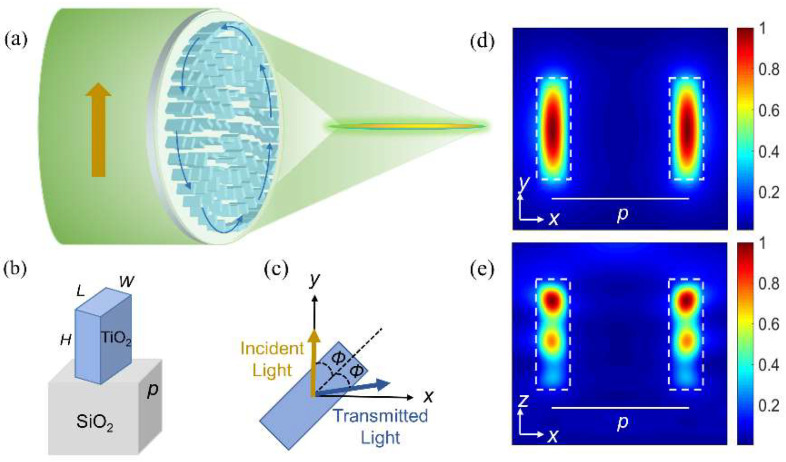
(**a**) Schematic of the needle beam generator based on the PB metalens. The yellow and blue arrows show the polarization directions of the incident or transmitted light, respectively. (**b**) Schematic illustration of a nanobrick of TiO_2_ sets on a substrate of SiO_2_. *L* and *W* represent the length and width of the nanobrick, respectively. (**c**) Each unit cell acts as a local half-wave plate. (**d**,**e**) The magnetic energy density of x–polarized light at the x–y cross−section above the TiO_2_ interface 0.5 μm and x–z cross−section, respectively. The white dotted line indicates the boundary of the nanobrick (parameters: *L* = 250 nm, *W* = 95 nm).

The schematic diagram of the generator of the needle beam is shown in Figure 2a. The PB metalens can convert the incident LPB (y−polarized) to APB, accompanied by vortex form and light focusing. The building blocks of the metalens are composed of TiO_2_ nanobricks and SiO_2_ substrates, as shown in Figure 2b. Dielectrics were chosen due to their high transmission efficiency compared to metals. The refractive indices of TiO_2_ and SiO_2_ were 2.46 and 1.46 at 532 nm, respectively. In order to prove the capability of local field manipulation by unit cells, we utilized the finite difference time domain (FDTD) method to calculate the magnetic energy density of nanobricks, as shown in Figure 2d,e. Because of the high refractive index contrast between the nanobrick and its surroundings, light energy is concentrated in the nanobrick, so that each nanobrick can be considered as a waveguide truncated on both sides. Thus, this leads to differences in the phase shifts (Φx and Φy) and effective refractive indices [18] of two waveguide modes polarized along *L* and *W* of each rectangle. Therefore, the LPB possesses a phase delay due to different velocities of polarization components along *L* or *W* of the nanobrick. Precisely because of this phase delay between the electric field components, the polarization of the transmitted beam changes. As a result, unit cells can work as local wave plates with an optical axis.

To realize the conversion from LPBs to APBs, these cells are required to act as half−wave plates (Figure 2c). When y−polarized beams propagate through these cells, transmitted light can be calculated using the Jones matrix method, as follows [27]:

(6) $$\left[\begin{array}{cc} \cos2\phi & \sin2\phi \\ \sin2\phi & -\cos2\phi \end{array}\right]\left[\begin{array}{l} 0\\ 1 \end{array}\right]=\left[\begin{array}{l} \sin2\phi \\ -\cos2\phi \end{array}\right]$$

In Equation (6), the Jones matrix denotes a half−wave plate with its fast axis rotated by an orientation *ϕ* with respect to the y-axis. The Jones matrix of the APB is ${\left[\begin{array}{cc} \sin\psi & -\cos\psi \end{array}\right]}^{T}$, which means that Equation (6) is the electric field vector of APB and *ψ* = 2*ϕ* (where *ψ* = arctan(*y*/*x*) is the azimuthal angle).

**Figure 3:**
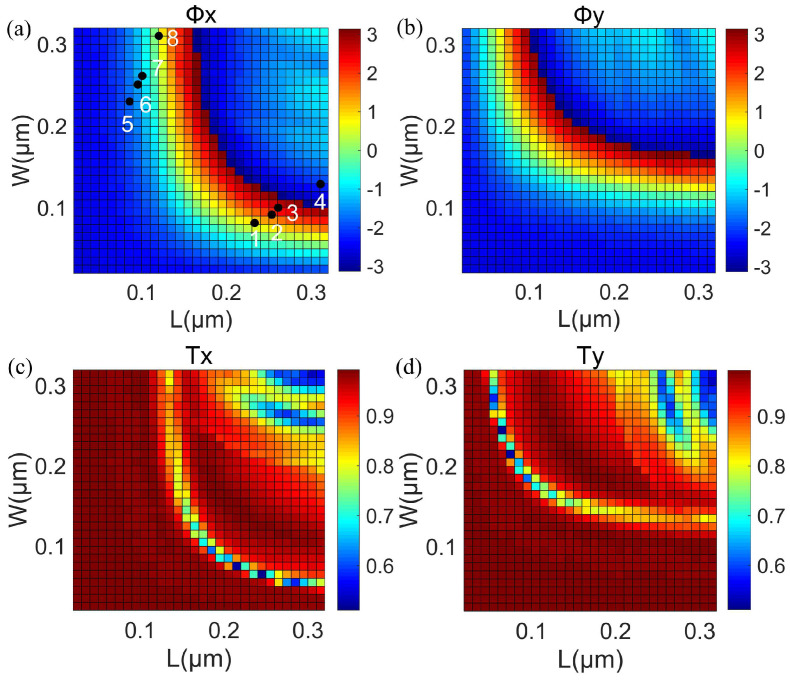
(**a**,**b**) For x− and y− polarized light incidences, phase shifts Φx and Φy are used as the function of the *L* and *W* of nanobricks, respectively. (**c**,**d**) The transmittance Tx and Ty are the function of the *L* and *W* of nanobricks for the incident x− and y− polarized light, respectively. The *L* and *W* of unit cells from 1 to 4 were *L* = 230, 250, 260, and 310 nm, and *W* = 85, 95, 100, and 130 nm. The unit cells from 5 to 8 were arranged by rotating the nanobricks from 1 to 4 at every angle of 90° clockwise.

Only when there is a π phase difference between the two linear polarizations aligned with *L* and *W* of the rectangle can each cell act as a local half−wave plate. To achieve this function in the metalens, a periodic array of nanobricks is illuminated by x− and y−polarized light at 532 nm to obtain the relationship between the sizes (*L* and *W*) of the nanobricks and phase shifts (Φx and Φy) as well as the transmission efficiencies (Tx and Ty). The *L* and *W* of nanobricks were both swept in the range of 20–320 nm while the height H and the period size p were kept at 600 nm and 370 nm, respectively. Periodic boundary conditions were set along the x− and y−directions of the nanobricks to avoid the influence of the interaction of neighboring unit cells. The mesh steps were 10 nm. As shown in Figure 3a,b, eight different nanobricks with a π phase difference between Φx and Φy were selected. Meanwhile, these nanobricks could maintain 0–2π phase coverage, and the phase increment of π/4 was maintained between the adjacent nanobricks. As shown in Figure 3c,d, the transmission efficiencies of the two incident beams were essentially larger than 90%, which ensures that the proposed metalens maintains a high transmission regardless of the rotation angle of unit cells. As a result, nanobricks can work as high–efficiency half–wave plates.

Based on the transmission phase, the metalens can add the vortex and lens phases to the beam. In order to make the designed metalens play the role in the loading vortex phase and focusing beam, the selected eight nanobricks were arranged according to the phase distribution in the following equation:

(7) $$\varphi (x,y)=\frac{2\pi }{\lambda }(\sqrt{x^{2}+y^{2}+f^{2}}-f)+m\arctan(y/x)$$

where *f* and *m* represent the focal length and topological charge, respectively. Here, *m* = 1. Based on the PB phase, the metalens can change the polarization of the incident light. In order to convert LPB to APB, the above eight nanobricks had a rotated angle of ψ/2 around their local position. Therefore, the azimuthally polarized vortex beam is expected to be generated and focused simultaneously.

## 3. Results and Discussions

### 3.1. Generating and Focusing the Azimuthally Polarized Vortex Beam with the Metalens

**Figure 4:**
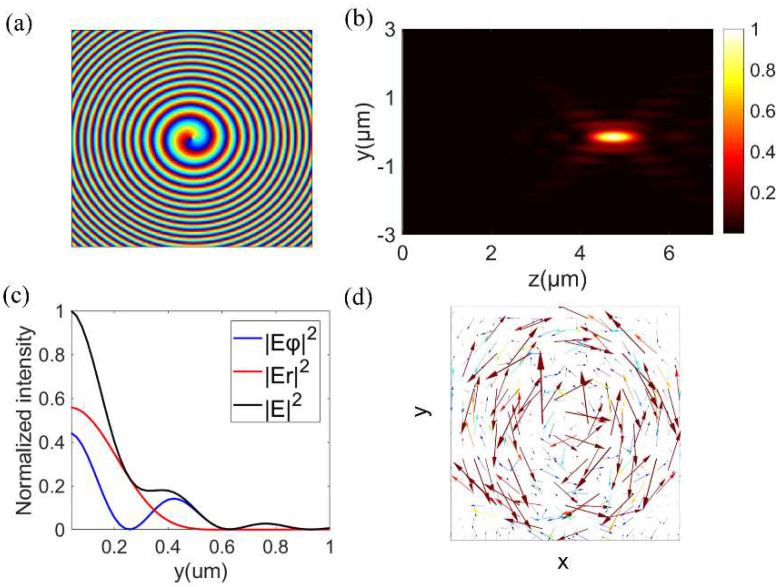
(**a**) The theoretical phase distributions of the designed metalens. (**b**) The intensity distribution in the propagating y–z plane of the focal field. (**c**) A radial normalized intensity distribution in the focal plane. (**d**) The polarization distributions of the x–y plane near the metalens (z = 100 nm).

With the above designed metalens, we simulated the intensity distributions of the electric field in the propagating and focal planes (Figure 4b,c) by the FDTD method. In the following simulations, the radii *R* and focal length *f* of metalenses were 10 μm and 4.84 μm, respectively, NA was 0.9, and the mesh steps were 20 nm in the x− and y−directions, but in the z−direction, they were 30 nm. A perfectly matched layer was set along the x−, y−, and z−directions. In Figure 4b,c, the FWHM and DOF of the focal spot were 0.55 λ and 1.9 λ, respectively. The intensities of the simulated radial and azimuthal electric field components in Figure 4c were not zero when y = 0, indicating that both of them had bright focuses, which is consistent with the phenomena in Figure 1a, but different from the doughnut-shaped focuses of the APBs. Furthermore, in combination with the phase distributions of the designed metalens and the polarization distributions of the transmitted light shown in Figure 4a,d, the proposed metalens may successfully convert a LPB into an azimuthally polarized vortex beam and focus the transmitted light simultaneously.

### 3.2. The Needle Beam Generated by BOE with Metalens

According to Figure 1b,c in the theoretical calculation, the BOE can not only reduce the size of the focal spot, but also extend DOF, so we loaded the phase distributions of BOE onto the metalens to produce the needle beam. After the phase distributions of BOE are loaded, the phase distributions of the metalens become:

(8) $$\varphi =\varphi (x,y)+\varphi_{BOE}$$

**Figure 5:**
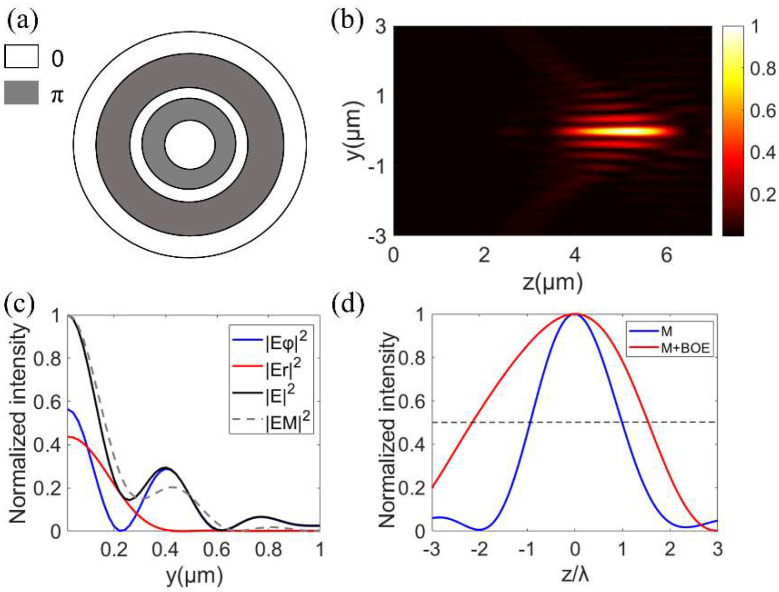
(**a**) Phase distributions of a five−belt BOE in the x–y plane. Phases in the white and gray areas were 0 and π, respectively. (**b**) The intensity distributions of the needle beam generated at the propagating y−z plane. (**c**) A radial normalized intensity distribution in the focal plane. |E_M_|^2^ represents the normalized intensity of the total focal field of the beam focused only by the metalens. (**d**) Represents the normalized intensity profiles along the z−axis. M and M + BOE represent the metalenses without and with the BOE phase focused beam, respectively.

The multi−region BOE used in this work is shown in Figure 5a. The total electric field distribution of the generated needle beam at the propagating plane is shown in Figure 5b. As can be seen from Figure 5b–d, the DOF and FWHM of the optical needle produced in this way were 3.42 λ and 0.47 λ, respectively, and both results were better than the results of focusing an azimuthally polarized vortex beam by the metalens only, which is consistent with the results in Figure 1b,c. Therefore, it can be considered that the single−layer metalens decorating phase distributions of BOE can generate an optical needle. Interested readers may refer to the supplementary document for further study of the results and discussions on the polarization evolution of the generated optical needle.

### 3.3. The Needle Beam Generated by an Annular Aperture with Metalens

**Figure 6:**
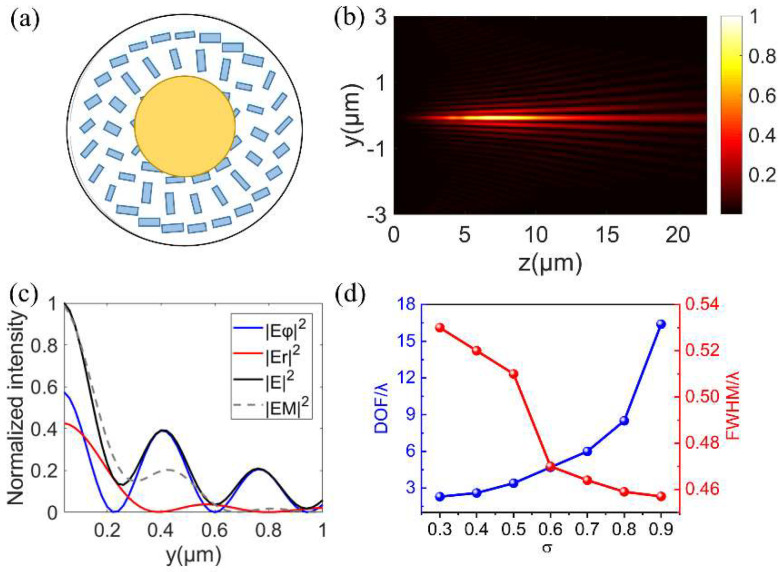
(**a**) The schematic diagram of the proposed metalens with an inner opaque gold plate. (**b**) The intensity distribution of the needle beam generated at the propagating y−z plane. (**c**) A radial normalized intensity distribution in the focal plane. (**d**) DOF and FWHM at different annular apertures.

According to the theoretical results in Figure 1b,c, the annular aperture can help to extend DOF and form a sharper focus. Then, an annular aperture is introduced to work with metalens to generate the needle beam. As can be seen from Figure 6a, the metalens had an inner opaque gold plate to form the annular aperture σ. The total electric field distribution at the axial cross−section is shown in Figure 6b. Obviously, the DOF of the focal spot obtained by this method was significantly longer than that in Figure 4b, which is consistent with the theoretical calculation results in Figure 1c. In combination with Figure 6b,c, the needle beam with a FWHM of 0.46 λ and DOF of 16.4 λ was obtained under σ = 0.9. In this method, the phase, polarization, and amplitude are controlled simultaneously to achieve an optical needle with subdiffraction size and long DOF. Interested readers may refer to the supplementary document to further study the results and discussion on the polarization evolution of the generated optical needle.

To show the impact of σ on focusing, we studied the opaque metal plate with different radii but a fixed R of 10 μm. The DOF and FWHM varying with σ are plotted in Figure 6d. With the increase in σ, DOF gradually extends and FWHM becomes smaller, and these two characteristics of the needle beam are improved. However, in terms of the property of the needle beam, it does not mean that the bigger σ is, the better. Because the electric field intensity of the side lobes of the needle beam also increases with the increase in σ, it is effective to obtain optical needles using the annular aperture, but this method is not efficient and can be optimized by a higher−order mode with multi-zone binary phase pupil filters [28].

## 4. Conclusions

In conclusion, we proposed dielectric metalenses based on the PB capable of generating and focusing an azimuthally polarized vortex beam simultaneously. It was proven that the proposed additional phase or amplitude modulation working with a metalens can achieve optical needles with narrow FWHM and extended DOF. The FWHM and DOF of the focal spot of the azimuthally polarized vortex beam were 0.55 λ and 1.9 λ when focused by the metalens only. The optical needle with a FWHM of 0.47 λ and DOF of 3.42 λ was generated by loading the phase distribution of BOE to the metalens. By combining the annular aperture and metalens, an optical needle with a DOF of 16.4 λ and subdiffraction FWHM of 0.46 λ could be obtained. Through the capability of metalens to modulate the amplitude, phase, and polarization at the same time, an optical needle with a subdiffraction size and long DOF can be generated. These efficient and flexible methods to obtain needle beams can potentially benefit applications in super-resolution imaging, photolithography, and particle trapping.

## Data Availability

Data are contained within the article.

## Supplementary Materials

The following supporting information can be downloaded at: https://www.mdpi.com/article/10.3390/nano12224074/s1, Figure S1: The cross-section of the ellipticity of the local polarization ellipse of the optical needle generated by the BOE and annular aperture (AA) combined with the metalens, respectively. Reference [29] is cited in the supplementary materials.
