# Supplementary material for: Generation of Subdiffraction Optical Needles by Simultaneously Generating and Focusing Azimuthally Polarized Vortex Beams through Pancharatnam–Berry Metalenses
Source: Nanomaterials (Basel). 2022 Nov 19;12(22):4074. doi: 10.3390/nano12224074 (PMC9693032; doi:10.3390/nano12224074)
Supplement: Supplementary file 1 [file nanomaterials-12-04074-s001.zip › nanomaterials-2020700-supplementary.pdf]

nanomaterials

MDPI

Supplementary Materials

Generation of Subdiffraction Optical Needles by Simultaneously Generating and Focusing Azimuthally Polarized Vortex Beams through Pancharatnam–Berry Metalenses

Zhe Shen* and Shijie Huang

School of Electronic and Optical Engineering, Nanjing University of Science and Technology, Nanjing 210094, China
* Correspondence: shenzhe@njust.edu.cn

The polarization evolution of the generated needle beam

The polarization evolution of the needle beam was studied by calculating the Stokes polarization parameter [S1], from which the ellipticity tanχ can be derived:

$$\sin 2\chi = i(E_x E_y^* - E_y E_x^*)/(E_x E_x^* + E_y E_y^*)\,, \tag{S1}$$

where E_x* and E_y* are the complex conjugate of E_x and E_y.

The polarized distribution of the needle beam can be analyzed through the ellipticity of the local polarization ellipse on the focal plane shown in Figure S1. Taking the case of BOE with metalens as an example, it is obvious that the polarization is spatially variable. Theoretically, the ellipticity of the pure transversely polarized needle beam is either 1 or −1 at the center of the focal plane because its central focal field is strictly circularly polarized. However, in our work, the ellipticity in the center of the focal plane is greater than −1, indicating that the focal field is left−handed elliptic polarization. In addition, the curve of ellipticity in the figure is asymmetrical. Those may be due to the slightly different conversion efficiency of unit cells of the inhomogeneous metalens. As the radial distance increases, the ellipticity changes rapidly between 0.8 and −0.8, so the handedness of the beam also changes between the left- and right-handed polarization. There are also some critical points, namely the point of ellipticity of 0, which indicate that only one component of radial or azimuthal polarization exists. Interestingly, the wavefront polarization of such beam is parallel to the transversal polarization of tightly focused circularly polarized light [S1] in the focal plane. For the former, the beam is completely transversely polarized, while for the latter, besides the transversal component, there is a significant longitudinal electric field component.

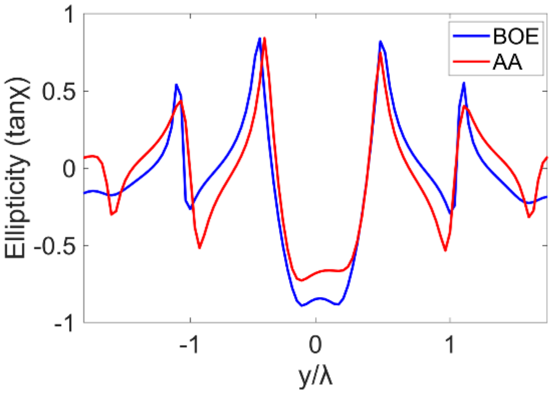

Figure S1. The cross-section of the ellipticity of local polarization ellipse of the optical needle generated by the BOE and annular aperture (AA) combined with the metalens, respectively.

Reference

S1. Bomzon, Z.; Min, G.; Shamir, J. Angular momentum and geometrical phases in tight-focused circularly polarized plane waves. *Appl. Phys. Lett.* **2006,** *89*, 241104.
